# How policy makers can support primary eye health care

**Published:** 2022-03-01

**Authors:** Luke Allen, Matthew Burton

**Affiliations:** 1 Clinical Research Fellow: International Centre for Eye Health, London School of Hygiene and Tropical Medicine, London, UK.; 2 Director: International Centre for Eye Health, London School of Hygiene and Tropical Medicine, London, UK.

**Policy makers play a vital role in ensuring that eye care services – including health promotion – can be delivered as an integrated part of primary health care.**

**Figure:**
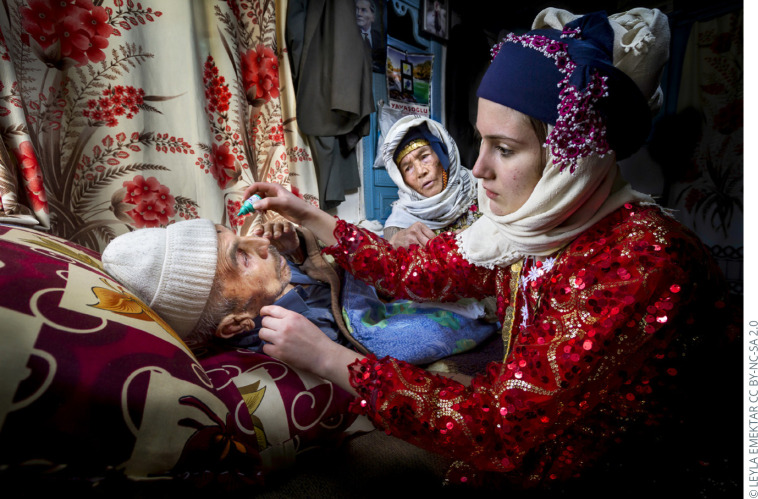
Policy makers can help to ensure that essential eye medicines are available to all. **TURKEY**

Primary eye health care (PEHC) is eye care that is delivered by frontline and community-based workers at local primary health care clinics and includes health promotion and multisectoral engagement. Policy makers play a vital role in ensuring that all of the correct ‘building blocks’ of the health system are in place so that eye care services – including health promotion – can be delivered as an integrated part of primary health care:^1^

1. A well-trained health workforce
2. Functioning health information systems
3. Access to essential medicines and supplies
4. Financing
5. Leadership and governance.

Each of these building blocks requires the involvement of policy makers at different levels within the health system:

- **International.** Policy leads at WHO and IAPB. They are responsible for setting global norms.
- **National.** Ministers of health, civil servants, and national eye care leads. They are responsible for strategic planning, allocating resources, and providing supportive structures for local service delivery.
- **Regional.** Commissioners, regional health department leaders, and city councillors are responsible for organising resources efficiently and equitably.
- **Facility:** Health facility managers are responsible for coordinating the delivery of high-quality care.

We will look at each of the building blocks in turn.

## 1 Health workforce

The people responsible for delivering eye care at primary level must be able to identify and manage basic conditions that are common in children, adults, and the elderly. In some countries, PEHC is delivered by optometrists, refractionists, and ophthalmic nurses, but in others, or in rural areas without eye care professionals, primary eye health care needs to be delivered by general primary level clinic staff, e.g., nurses, community health workers, and medical officers who look after people over time, as they grow and age.

Ideally, primary eye care workers would also be responsible for health promotion and disease prevention (see article on health promotion in this issue). They are well placed to counsel individual patients, but also build relationships with the local community and work with them to see how the unique population-level risk factors that drive eye disease in the local area might be addressed. Primary eye care workers require time, training, and resources for this – which must be facilitated by policy makers at national and regional levels.

National policy makers should design curricula and minimum competencies based on international PEHC norms and standards, set by the World Health Organization (WHO) and other international eye agencies, to ensure that all citizens are able to access the eye care they need. Supervision structures are required in order to support high quality care. As the Nigerian case study in this issue illustrates, it is also important that policy makers consider the skill mix required to deliver high quality care to local communities and align pre-service and in-service training.

## 2 Health information systems

National, regional, and facility-level policy makers have a role to play in ensuring that staff members delivering PEHC have access to adequate medical records and functioning IT systems. The best information systems will allow all the professionals involved in a patient's care to see the records made by each other as well as the background medication, risk factors, and other medical problems. The advantages are doing so include:

- More coordinated, patient-centred care
- Less duplication of work
- Reduced potential for medical errors
- Better longer-term follow-up, when needed.

To support the planning of services, policy makers should also routinely gather and analyse data.^2^ The data can come from two main sources:

1. Epidemiological surveys on the prevalence of eye conditions, access to treatment, and outcomes of treatment.
2. Facility-based data, to assess local access to services.

Last, but not least, eye health indicators should be included in national reporting systems to ensure that progress is measured and so that funding can be allocated where it is most needed.

The Nigerian policy analysis in this issue recommends using eye care data as one of the core indicators of primary care performance. In Rwanda, indicators for eye services were included at the primary level in the country's performance-based financing scheme, which has contributed to staff member motivation (see Rwandan case study).

## 3 Access to essential medicines and supplies

National policy makers are responsible for setting the essential medicines list, in line with WHO recommendations. Regional policy makers are responsible for coordinating procurement and logistical support to ensure that all facilities are able to obtain essential medicines for eye health. Facility managers hold the primary responsibility for ensuring that their facilities are well stocked with in-date medicines, and that their staff have access to the basic equipment required to deliver high quality care. There is a need for alignment and coordination across government to ensure that policy recommendations are adequately resourced. To take a final example from the Nigeria case study, whilst the national newborn policy recommends using topical erythromycin for conjunctivitis, this product is currently not on the essential medicines list.

## 4 Financing

National policy makers are responsible for delivering Universal Health Coverage, which means universal access to essential health care services without imposing financial hardship.^3^ This involves raising and pooling funds through taxation or insurance schemes, and then distributing resources to service providers so that eye care services can be offered free at the point of use or for an affordable fee. As most countries have a mix of public, private, and charitable providers, regional policy makers need to ensure that local populations have access to affordable services. This includes access to high quality primary eye health care services. Policy makers will need to consider the unique burden of eye disease and visual impairment borne by their populations, and then calculate the resources required to deliver a core ‘basket of care’ to meet this need.

## 5 Leadership and governance

National and regional policy makers set national norms and standards, based on international best practice. They are also responsible for developing quality management systems to identify variation in clinical practices and then support regions and local services that are struggling to meet these standards of care. PEHC is a relatively novel concept that requires new governance systems to be established in order to set norms, develop appropriate national standards, and – for many countries – adapt their information systems so that they can monitor and manage quality standards.

## Summary

In order to do their job well, the people who deliver primary eye health care need training, information systems, medicines and equipment, financing, and standards and guidelines. Policy makers at the international, national, and regional levels all play important roles in ensuring that these elements are in place.
